# Qualitative Case Study: A Pilot Program to Improve the Integration of Care in a Vulnerable Inner-City Community

**DOI:** 10.5334/ijic.6184

**Published:** 2022-05-16

**Authors:** Margaret Frances Williamson, Hyun Jung Song, Louise Dougherty, Lisa Parcsi, Margo Linn Barr

**Affiliations:** 1 University of NSW, AU; 2 University of NSW and Sydney Local Health District, AU; 3 Sydney Local Health District, AU

**Keywords:** link worker, integrated care, health navigation, vulnerable populations, qualitative evaluation

## Abstract

**Introduction:**

There is a strong correlation between vulnerable populations and poor health outcomes. Growing evidence suggests that person-centred interventions using ‘link workers’ can support communities to navigate and engage with health and community services, leading to improved health service access. We describe the initial phase and qualitative evaluation of a Healthy Living Program, supported by a link worker role. The Program aimed to improve health service access for residents of an Australian inner-city suburb.

**Methods:**

To inform future program development, semi-structured interviews were conducted with clients and stakeholders (n = 21). The interviews were analysed thematically to understand program impact, success factors, constraints and potential improvements.

**Results:**

Key themes relating to impacts were a new model of working with community, improved access to services, and responsiveness to community need. Key factors for success included being a trusted, consistent presence, having knowledge of the community and health system, and successful engagement with the community and stakeholders. The constraints included difficulty influencing health system change and lack of community input. Suggested improvements were expanding the service, enhancing health system change and increasing community involvement.

**Conclusion:**

Knowledge gained from this study will inform future integrated approaches in health districts to address health inequities in areas of need.

## Introduction

In 2017, the Australian Health Policy Collaboration published a report [1] highlighting the growing health disparities in Australia which correlate closely with socio-economic status. The report showed that 40% of low-income Australians experienced poor health outcomes. It attributed these poor health outcomes to multiple factors including poor access to healthcare, poor nutrition, high rates of obesity and high smoking rates. These problems overlap social issues related to housing, poverty and inadequate education [2,3].

Addressing the health needs of populations experiencing disadvantage can be difficult, especially if their needs are complex and solutions to address these issues do not fit neatly within existing health systems and practices. Person-centred interventions using community health workers (CHWs), patient navigators (PNs) and link workers (LWs), can help to support the community in navigating and linking them with health and community services. A significant body of literature exists on interventions using CHWs, PNs and LWs in various settings to help vulnerable communities navigate complex health systems, improve service delivery and address the social determinants of health [2,4,5]. There is growing evidence that person-centred interventions provided by these roles, can be effective in improving access to health services (especially cancer screening) [4,6,7,8], promoting a wide range of healthy behaviours [2,4], improving chronic disease management [2,9,10,11,12], reducing preventable health service use [13] and improving the overall health and wellbeing of populations [12], including those from disadvantaged groups [2,6,9,14].

The success of these interventions has been linked to factors related to the planning and development of the programs, how the program is delivered, staff attributes, the accessibility of referral services, community engagement and the integration of the work within a supportive health system [15,16,17,18].

As health authorities attempt to address the health needs of disadvantaged populations using similar patient-centred community-based intervention models, an understanding of the factors that contribute to their success in specific settings and of the challenges for such programs, is important in order to enhance implementation and outcomes [15].

## Healthy Living Program

The target area for the Healthy Living Program (the Program) was Waterloo, an inner suburb of Sydney, Australia, with public housing representing over one quarter of the homes in the suburb (28%) and 90% of homes being flats or apartments [19]. The suburb has a number of vulnerable populations, including those who are economically disadvantaged, those from culturally and linguistically diverse backgrounds and those with complex social and health needs [19]. More than half of public housing residents are over the age of 60 years, 66% were born overseas, 8% have an Aboriginal and/or Torres Strait Islander background, 86% receive some form of pension or financial assistance.

Australia’s health system aims to provide safe, affordable and quality health care to all Australians. The federal government funds aged care services and subsidises access to community-based general practitioners (GPs), medical specialists, nurses; some allied health professionals and medicines. State and territory governments are responsible for public hospitals, community and mental health services, ambulance and emergency services and public health and preventive services. The Sydney Local Health District (SLHD) is one of 15 publicly funded Health Districts in the state of New South Wales and is responsible for the management and implementation of state-run health services and the health and wellbeing of the residents in this inner suburb (in addition to the needs of residents of other suburbs within the SLHD’s catchment area, which has a population of more than 670,000) [20]. The SLHD established the Program in 2017, in response both to community concerns about the range of health issues faced by the Waterloo community and perceptions that health services were not responsive to the needs of the community living in the Waterloo public housing estate [21]. The Program was implemented as the first phase (‘initiative and design’ phase) in the development of a broader integrated care model to improve access and integrate care among vulnerable communities within the SLHD [22].

The Program aimed to develop processes to: (1) better understand the health and wellbeing needs of the community; (2) provide navigation services to facilitate access to health services for individuals, groups and the community; (3) advocate changes to the way services are delivered to meet community need; (4) support community development activities to reduce health disparities and improve the wellbeing of the community; and (5) facilitate improved connectedness and communication between the community, other government and non-government organisations (NGOs) and SLHD services.

A Healthy Living Link Worker (the LW) was appointed to explore how program staff might act as a point of connection, liaison and navigation between residents and the SLHD services, and ultimately lead to improved service delivery and better health outcomes for residents. The work was supported by two independent advisory groups, one for SLHD managers and one for community and community-based NGO representatives. The Program had been in place for over two and a half years, and the most recent incumbent of the LW role had been employed for almost 12 months. Clients for the navigation services were self-referred or referred by local community NGOs or government agencies.

**Figure 1:**
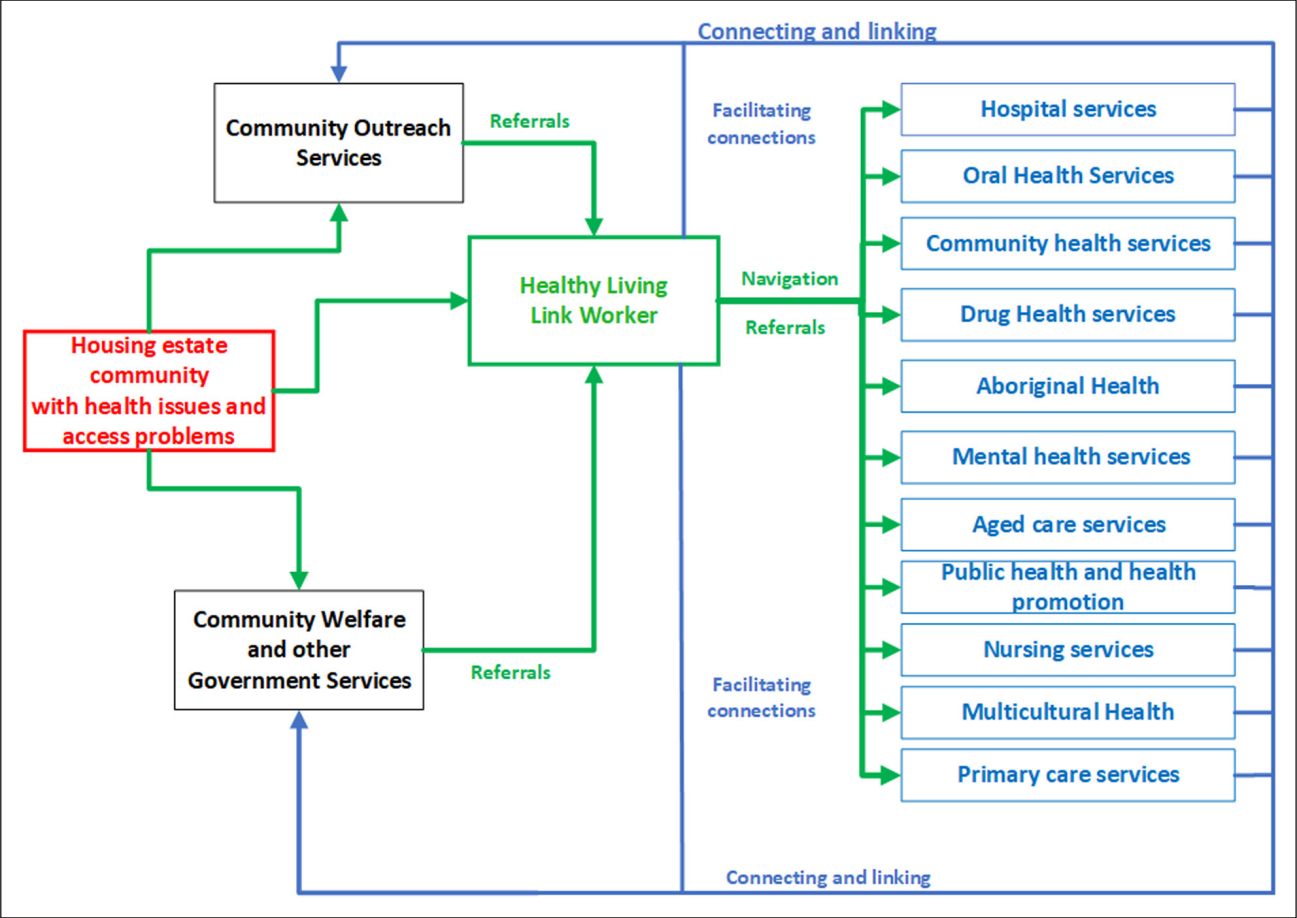
Overview of the service environment for the Healthy Living Program.

At the time of the evaluation, the LW had worked with 75 clients and associated SLHD staff to navigate appropriate pathways of care in the previous 12 months. Client information was collected by the LW and recorded in a restricted database, not connected to the SLHD clinical records. The LW also worked with specific SLHD services to improve access to urgent care for specific client groups, including those with chronic complex health needs. Part of the work included co-ordinating and supporting wellbeing checks, health information sessions and the delivery of health information to a range of at-risk community groups. Figure 1 provides an overview of the complexity of the work and the number of different services and referral pathways that need to be negotiated and integrated.

## Evaluation

We conducted a qualitative study as part of a larger evaluation of the Program. Semi-structured interviews investigated the perspectives of the clients, community-based NGOs and health and welfare government staff, on the perceived impact, success factors, constraints and potential improvements for the LW role.

Eighty key individuals who worked with or were affected by the role, were identified through discussions with the supervisor of the Program and the current incumbent. These key individuals included 30 clients who had directly interacted with the LW and were referred to or were involved in a LW initiated activity; and SLHD staff (n = 23) and staff from NGOs (n = 14) and other government agencies who provided services to the Waterloo residents and had worked with the LW. We limited interviews to those who were able to participate without assistance in the English-language interviews as the majority of the LW interactions and activities were in English.

**Figure 2:**
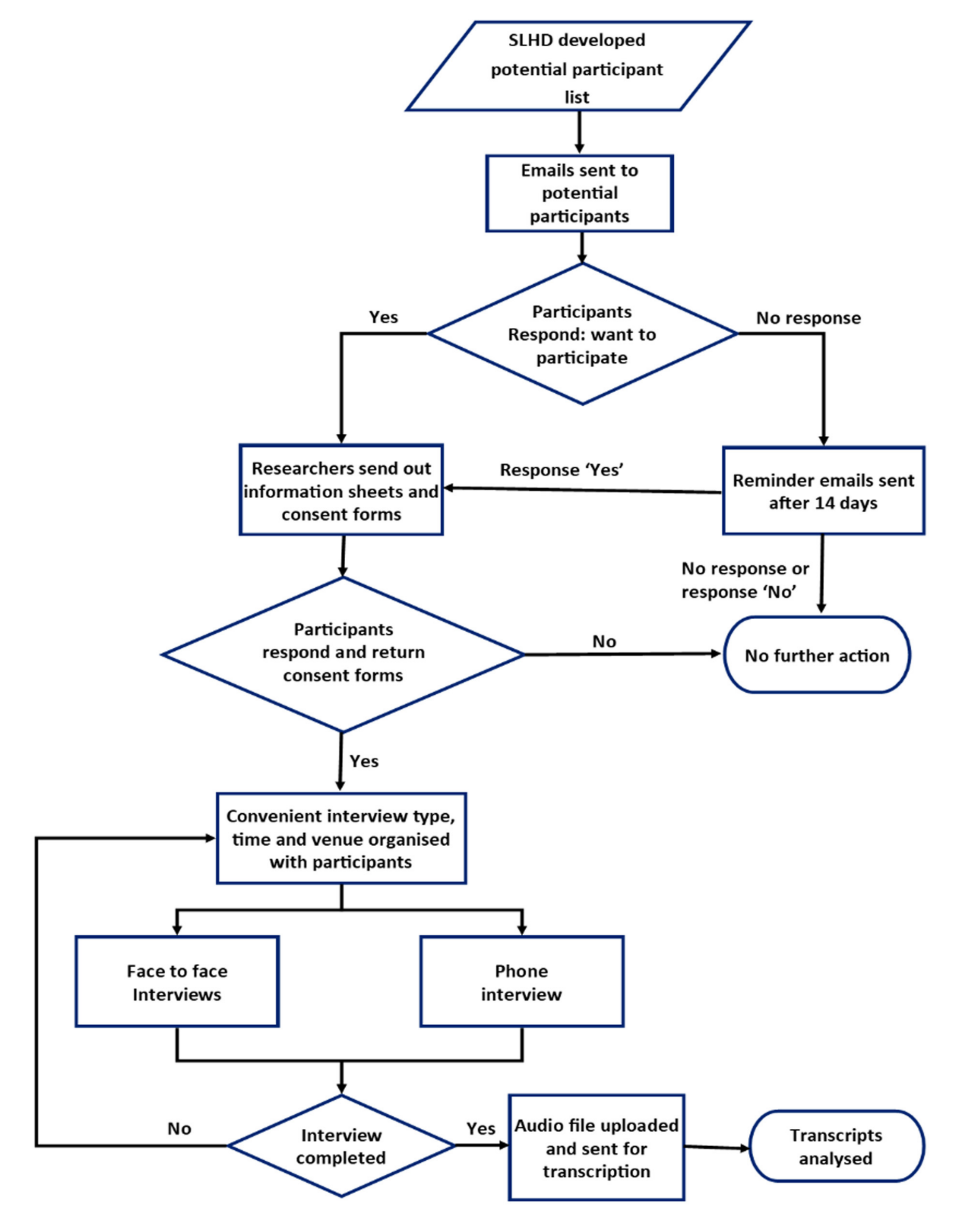
Recruitment and data collection.

We aimed to interview at least five individuals from each group to gain a wide range of experiences, views and opinions. The supervisor of the Program and/or the current incumbent sent email invitations to the potential informants, inviting them to participate in the interviews. Some community members without email addresses were contacted by phone. Interviewees gave written and verbal consent to participate. Figure 2 summarises the selection, recruitment and data collection methods.

### Data collection and consent

Preliminary discussions with key stakeholders and the current LW on the impacts and key success factors and challenges for the program, informed a semi-structured interview guide (Table 1). The questions focussed on the LW role and were adapted for community members and staff of the SLHD and other agencies. The research team pilot tested the interview guides for their comprehensibility, and questions were revised appropriately. Telephone and face-to-face interviews were conducted by two researchers between 9 February and 26 March 2020, and were recorded and transcribed verbatim.

**Table 1:** Interview guide.


COMMUNITY QUESTIONS	STAFF OF THE HEALTH DISTRICT AND OTHER RELEVANT ORGANISATIONS QUESTIONS

1. Can you please tell us how you got to know [the Link Worker]? Probe (if not addressed): Can you tell us about a specific time when [the Link Worker] has helped you or someone you know?	1. How have you (or other staff in your organisation) been involved with the Healthy Living Link Worker role? Probe: Is there a process or protocol which guides these interactions?

2. How well do you think [the Link Worker] works with people in the community?	2. How successful has the Healthy Living Link Worker been engaging with: a. the community b. the staff of your organisation c. the staff of other organisations such as local Health District, community-based non-government organisations, Families and Community Services or other government organisations? Probes: If yes – can you provide some examples? How has this been achieved? If no – can you provide some examples?

3. How is [the Link Worker] making a difference in the community? Probes: In your opinion, what are the most helpful activities of [the Link Worker]? What other activities could [the Link Worker] be involved in to help the community?	3. In your opinion, what has the role achieved? Probes: What has worked best? Why? What isn’t working? Why?

	For Local Health District staff ONLY: 4. What capacity does your service have to support those clients the Healthy Living Link Worker refers to you? Probes: If they have capacity – Can you provide some examples? If they don’t have capacity – What has been the issue(s)?

4. Overall, are you satisfied with the job that [the Link Worker] is doing?	5. Overall, are you satisfied with the activities of the role? Probes: How could the role function better? Are there any other activities the Link Worker could be involved in?

5. Do you have any further comments or suggestions about what [the Link Worker] is doing for the community?	6. Do you have any further comments or suggestions about the Healthy Living Link Worker role?


### Data analysis

The transcripts were thematically analysed through an iterative process. The research team met regularly to discuss coding and analysis, identify themes and resolve any disagreements or concerns [23,24,25]. Based on a sample of the transcripts, two researchers developed and discussed the initial coding framework with the research team. Themes were refined as more interviews were analysed, and they were further refined with agreement from the team. NVivo 12 software [26] was used for coding and analysis.

### Ethics approval

Ethics approval for the study was granted by Sydney Local Health District Ethics Review Committee (RPAH Zone) X19-0357 and 2019/2019/STE16400.

## Evaluation Findings

Of the 39 individuals contacted, 21 agreed to participate (participation rate = 54%). The characteristics of the interviewees are presented in Table 2.

**Table 2:** Interviewee characteristics.


CHARACTERISTICS		NO OF INTERVIEWEES (%)

**Group**	Clients	7 (33.3%)

	NGO staff	4 (19.0%)

	SLHD staff	6 (28.6%)

	Staff from other government agencies	4 (19.0%)

**Gender**	Female	16 (76.2%)

	Male	5 (23.8%)

**Age-group**	30–49 years	12 (57.1%)

	50–69 years	9 (42.9%)


Thematic analysis focussed on the four main areas of investigation: impact, success factors, constraining factors and suggestions for improvements for the Program and the LW role. The themes identified for each area of investigation are presented in Table 3. The clients interviewed had previously sought the help of the LW to access services, and their responses during the interviews were mainly concerned with the impact of the role and the success factors in terms of their own experience. Sub-themes are bolded in the text.

**Table 3:** Themes according to areas of investigation.


**PERCEIVED IMPACT OF THE ROLE**

New model of working with the community

Directing and supporting access to services

Responsiveness to community needs

Facilitating changes in community attitudes to services

Facilitating change in service delivery and collaborations

**SUCCESS FACTORS**

Trusted and consistent presence

Communication and personal skills

Knowledge about the community and the health system

Successful engagement with the community and stakeholders

**CONSTRAINTS OF THE ROLE**

Too much for one person to fulfil

Different expectations of the role

Lack of clarity about the role

Difficulty influencing health system change

Lack of community input into the work

**SUGGESTED IMPROVEMENTS**

Expansion of the service

More support to influence system change

Address other outstanding health service gaps

Need for the community and NGOs to be more actively involved


### Perceived impact of the role

Interviewees identified that the LW had **developed a new model for addressing health issues in the community**, was a dedicated presence in the community, listened to their issues and worked with others to address the health needs of the community.

“This is not a model of care that we’ve done before. … So this is actually a really important service, in my opinion, because of actually doing it in a different way. It’s actually looking at a community and saying, ‘What are its needs?’, and actually going out and meeting people and engaging with people in that local area and thinking about how do we do things better.” (SLHD staff 04)

“I think the community would have benefited a lot … their concerns have been tackled, somebody did something. And I think that means a lot.” (SLHD staff 02)

Social isolation, mental health and oral health of children and youth, were found to be significant problems for the residents of the Program target area. The LW had partnered with NGOs and other government agencies to support a community choir to reduce social isolation, improve mental health and build community connections and skills for the local community.

“[The choir] has been a really fantastic initiative. Yeah, it’s been really positive and a lot of work, … I think it had some really great benefits for people who have been coming along regularly.” (NGO staff 01)

The LW also collaborated with the local dental hospital to create a systematic change in the way pre-school children and young people with complex needs accessed urgent and preventive oral health services.

“[The LW] highlighted that the youth health was an area which was having issues linking with oral health.” (SLHD staff 06)

A range of interviewees identified that a key impact of the role was **directing individuals to appropriate health services** and supporting their interaction with service providers.

“I have a problem with [health condition] and needed surgery. And [the LW] helped me to get to the hospital. I was very sick.” (Client 01)

“Having a go between, you know, someone to talk to the authorities, you know, and the little guy, that’s us, it always helps, you know, because you don’t know where to begin and … [the LW] has a foot in the door, has a fair idea what direction to take. [The LW] helps us.” (Client 05).

Almost all clients were satisfied with the assistance provided by the LW.

“I am completely and honestly satisfied with his job for other people because I can see … when we first meet, I am very sick and have no family, [the LW] helped me.” (Client 01)

Several interviewees conveyed the importance of the navigation services provided by the LW in **addressing community need**.

“The big achievement I think is to make a health network accessible to the community, and it might seem to be a small thing to say, but it means a lot. It means a lot to the community, it means a lot to us as well.” (Other govt staff 04)

“I think navigation is his key strength. It’s navigating services for the vulnerable population.” (SLHD staff 06)

The respondents reported that access to the LW had led to **individuals changing their attitudes to health services** and being more engaged with health services.

“And I have seen some massive outcomes that come back with positive results … a lot of the clients in Waterloo and Redfern are not being retraumatised within the health system. It seems that re-traumatisation seems to be decreasing. A lot of them are willing to readily engage back with health services.” (NGO staff 02)

And we’ve noticed that because [the LW] has been involved, the patient’s attendance rate has been higher.” (SLHD staff 02)

Generally, respondents reported that the LW **facilitated collaborations** between stakeholders within the SLHD, NGOs and other government agencies, enabling the community to connect with the required services and supporting activities with other agencies to improve the health and wellbeing of the community.

“And I’m glad, because we were able to do something for that community that really needed that help. And had [the LW] not referred them, they would still be in pain, and waiting, and leaving it for later.” (SLHD staff 02)

“[The LW] has brought in other health organisations like Diabetes Australia and Drug Health and Mental Health to different community events, and Multicultural Health.” (NGO staff 02)

“I think there has been a couple of things that have really worked quite well, and one of them was the Health Expo that was held, in terms of getting a range of health organisations together.” (NGO staff 04)

### Success factors for the role

Respondents reported that the LW was a **trusted and consistent presence in the community** and on committees where [the LW] represented the SLHD. The LW’s ability to build trusted working relationships with community members was valued by the community and the NGO staff.

“[The LW’s] got the trust [of the community].” (NGO staff 01)

“I think having the same person in the role, over a consistent period, and where that position has been routinely reliable, that makes all the difference to that engagement, that relationship building …” (NGO staff 02)

“You can call [the LW] anytime and [the LW] responds straight away. Anytime! Anytime! [the LW] checks if you are feeling good or OK! It is a good service.” (Client 01)

The LW’s ability to **communicate and connect** with community members and staff from a range of organisations was reported to have facilitated the work of the role.

“[The LW has] always been good with talking to people and that, you know, communicating and stuff.” (Client 04)

“With me, [the LW] understand [sic] my position, so what I’m going through. So for me I think [the LW] is doing good.” (Client 06)

“[The LW] listens to me and tries to establish what’s the best way.” (Client 05)

“[The LW] really knows how to build this connection, it comes naturally. [The LW’s] a people’s person … [the LW] can talk to you about a very difficult subject, … [the LW] really knows how to make you at ease, and that’s one of his really big positive attributes.” (SLHD staff 02)

A key factor for the success of the role was **knowledge of the community and the health system**.

“I think they’ve got someone in the role who has a depth of knowledge and a connection with that particular community that is hard to replicate really.” (NGO staff 01)

“You know, [the LW] can organise things, [the LW] knows. [The LW’s] really knowledgeable about what services there are and things like that.” (Client 04)

Almost all interview respondents reported the LW had **successfully engaged** with specific groups in the community, including housing estate residents and Aboriginal and Torres Strait Islander peoples.

“Good links with the Aboriginal community, which is important, as it’s a high Aboriginal population in Waterloo … Seems to be good engagement.” (SLHD staff 03)

“The LW participated in the weekly outreach sessions [at the housing estate] that have happened, so that’s a way of being able to, sort of, make direct contact with people who have health concerns.” (NGO staff 04)

They also stated the LW supported collaborations within SLHD and between the SLHD services and the community and other organisations. This **engagement across sectors** was thought to contribute to the role’s impact.

“The Health Expo …… a lot of community members came, there were a lot of services represented, so people could engage with a number of both [SLHD services and other government services].” (SLHD staff 03)

“I think the LW’s engaged very well with Aboriginal health staff, I think that’s worked really well, and there’s been a bit more health promotion going on there.” (SLHD staff 04)

“And [the LW] went out of his way to engage on our behalf and speak on our behalf … we were getting almost no consents and dropping off that site in – from our project, working with [the LW], we got a really good response.” (SLHD staff 06)

### Constraints of the role

Five themes emerged related to constraints of the role. Three of the themes focussed on the function of the role. The position description stated that the role had three main functions: navigation, systems influence and capacity building. Some respondents felt that the responsibilities for each of these functions were **too much for one person**.

“One person can’t do everything.” (NGO staff 03)

“It’s probably identified as too big a job for one person. I think it’s identified that there’s different components to the job as well as different specialities.” (NGO staff 02)

Various SLHD and NGO staff had **different expectations of the role**. Some questioned whether the focus of the intervention work should have been at a patient level or at a system level. Several interviewees suggested that the role could have a more strategic focus.

“I think there’s probably a disconnect between what’s anticipated from the role and what the role can actually achieve.” (NGO staff 01)

“I think there’s still confusion about whether it’s strategic level or whether it’s patient level, and, I think, people are saying it’s both, but it’s a stand-alone position … I would have seen it as a strategic role … It’s not a community health worker role, I didn’t think. There is a link, but I think it should be more strategic.” (SLHD staff 05)

Interviewees from the NGOs were concerned that although they had been instrumental in setting up the role, there was a **lack of community input** in the direction of the LW’s work.

“….. One of the things that happened with this role, was when it started, the NGOs were involved in calling for it, were meeting with senior staff about the role quite regularly, and quite frequently. And that all stopped when the role came into play.” (NGO staff 02)

### Suggested improvements

A number of key informants from SLHD, NGOs and other government agencies, suggested the value of **expanding the LW work** by increasing the availability of the LW’s role and employing additional staff in the same role or with different skills, with a focus on the navigation work.

“Like, all of that foundation is there. I think it’s just about expanding it. That would be my one thing, that I would love to see more of it … I think with more resources or another person, I think that a lot more could be achieved.” (Other govt staff 02)

“I think with the district that [the LW’s] covering, there needs to be … maybe between one or two staff underneath [the LW] to cover the area that [the LW’s] covering, because it is quite diverse and quite complex.” (NGO staff 03)

“If [the LW] had a couple of people working with [the LW] in a team, that would be great, wouldn’t it?” (NGO staff 01)

Some key informants made suggestions about how the role could more strategically **influence local health system change**, including developing more structured working relationships between the LW and SLHD services, permitting the LW to have “authority to negotiate with services to bring about change”, and finally, empowering health services to take a more holistic approach and work together to address community health needs.

“I think that role has a capacity to sit at a broader more systemic level and coordinate, I guess, change at that level.” (Other govt staff 01)

“I think there’s opportunities where [the LW] could be involved, and I think that comes back to that strategic level where, if you make the relationships between the other services and look for gaps or identify opportunities at that level, I think, there’s an element of that missing.” (SLHD staff 05)

“What would be ideal I think would be [if the LW] can negotiate the high-level kind of, how to improve the system, like the navigation, the systems navigation … but also, some way to influence the health side of the equation more.” (NGO staff 01)

Some key informants suggested **outstanding health service gaps** that should be addressed, including support for housing estate residents caused by the uncertainties related to the redevelopment of the housing estate, and more support for residents with acute and ongoing mental health conditions.

“… Given the current state of the community with the [housing estate] redevelopment, … I think the anxiety and the mental health issues around that, there would be potential to do some workshops or some sort of regular support groups that could support the community because it has been an extremely stressful time.” (Other govt staff 02)

“Stuff around building resilience in the community, before the moves, before the relocations, would be great.” (Other govt staff 04)

“… Mental health is a major concern. Within social housing, … they just can’t get the access they need immediately … They’re just not getting the right support and services that are needed.” (NGO staff 03)

Interviewees from NGOs also identified the **need for the community and NGOs to be more actively involved** in ‘steering’ and supporting the work of the LW, and for ongoing consultation with the community. This included creating a steering group or reference group, with representatives from the community, NGOs and the SLHD working together to detect issues and address them.

“… We would have preferred the structure for the reference group to have the health people involved and the non-government people [community and NGOs] involved to have been in the same group … that increases the understanding of everybody around the table, and quite often it also means that people in power will also understand that there are aspects to a problem that they might not necessarily be aware of.” (NGO staff 04)

## Discussion

Our case study describes the initial phase of a program designed to identify and address health needs and health care access for community members from vulnerable groups in an inner-city suburb. This component of the program evaluation, which focussed on the work of the LW and was based on interviews with community members and staff from the SLHD and other relevant community organisations, explored perceptions on the impact of the work, its success factors and challenges, and provided suggestions for how the LW role and the broader Healthy Living Program may be improved. Overall, the interview respondents reported that the LW was working successfully with individuals to identify their health needs and to find ways of addressing access and service delivery issues. The work also addressed some of the health issues of the broader community, including the oral health needs of residents, acting as an advocate or link, and enabling changes to the way health services were delivered to meet community need for individuals and some at risk groups.

The interviews with staff from NGOs, other government agencies and SLHD, highlighted factors which have facilitated the success of the role. Having the relevant experience and personal skills, such as being a good communicator and having knowledge about the community and health services, were critical factors for success and acceptance of the LW as a trusted, respected and consistent presence in the community. A number of reviews and studies of similar roles, such as CHWs, LWs and PNs, have found similar factors as facilitators of success in other vulnerable groups [5,7,15,16,17,18,27,28,29,30].

The interviews highlighted the importance of the LW’s ability to communicate with and effectively promote collaboration with service providers and stakeholders, including coordinating their involvement in activities to promote health. Similarly, Pescheny and colleagues have noted the value of effective and regular communication between LWs and service providers in facilitating service delivery [30]. A range of other studies have also reported good communication between these stakeholders as an important enabler for improving integrated service delivery [7,15,16,30]. Trust was seen as a vital element for acceptance and connection to the clients within the community of similar programs [5,7,16,27,28], especially among marginalised populations [7]. One factor that was not emphasised in the literature but which both clients and staff from other government and community organisations valued in this study, was the importance of the LW being a consistent presence, whether at the end of the telephone for support, at meetings or through ongoing involvement in projects valued by the community and other stakeholders.

Constraints of the role which were highlighted in the interviews, such as the number and range of responsibilities of the LW and the lack of role clarity, matched the available information in the literature. In their reviews of the factors influencing implementation and maintenance of navigation programs, a number of studies attributed role clarity for the worker as well as for clients and health service providers, to the success of the program [5,7,15,17,18,29]. A review of an indigenous health worker program revealed that a good understanding of their role by service providers and Aboriginal community members, was an enabler in effective care coordination [18]. In addition to a lack of clear understanding and agreement on the LW role, disengagement by health service providers and a lack of shared partnerships between navigator services and other stakeholders, were found to be major barriers to implementation in a review of social prescribing services [30]. Perhaps as this Program was in the early stage of development, and although the need for the Program was identified, the scale of the demand for assistance led to interviewees suggesting the work was too much for one LW and that additional staff be employed to cover the shortfall.

This qualitative evaluation also raised other barriers, including the lack of ownership of the work by community members and community-based NGOs, and the difficulty in bringing about changes to how health services were delivered. This issue was also explored by Scott and colleagues in their review of reviews on CHW programs, which revealed two key enablers for the effectiveness of these programs in effecting system change: community embeddedness and integration into the health system [29]. Achieving community embeddedness was tied to active involvement of community members in CHW recruitment, priority-setting and monitoring. Integration into the health system would ‘foster’ respectful collaboration and communication between workers and senior health staff, and the health service may benefit from the ‘unique and practical knowledge’ of the workers. This in turn may lead to system change.

The WHO guideline on health policy and system support to optimise CHW programs, made similar recommendations related to the constraints identified in our evaluation, including: the necessary personal attributes and professional experiences of the workers; adequate funding; a clear understanding of the scope of work and its anticipated responsibilities and role by the workers, clients and stakeholders; community engagement, including community participation in the selection of workers, priority setting and monitoring of the work; and integration in and support by the health system [17].

Following our evaluation, five key high-level recommendations were made to the SLHD: investigate opportunities to extend the work; concentrate the work on significant issues faced by the community; increase community involvement in the program of work; improve collaborations between the Program, individual SLHD services and the community; and link the Program with other similar programs within the SLHD or wider. The SLHD currently is considering the recommendations as it develops the next phase of the Program.

The main limitation of the interview study was that there may have been people who were not interviewed who had very different views to those who were, including community members who could not communicate without assistance in English. However, the study did capture interesting and pertinent views from a wide range of stakeholders, and similar themes emerged across the diverse groups, suggesting that most of the important issues perceived by the participants were identified. These themes also aligned with the literature.

## Conclusion

Many health jurisdictions around the world are looking at ways to address the social determinants of health in their vulnerable populations and improve the integration of their health care. We present the first phase in the development of a Healthy Living Program, this being the implementation of a LW in the community, as part of an integrated care initiative in an inner-city suburb with a large housing estate. The Program aimed to address community concerns about a range of health issues faced by the community and community perceptions that health services were not responsive to their needs. Our evaluation showed that this new model for addressing health issues in this community was well received and respondents reported that it provided a valued link between the community and the health services. While most clients and stakeholders were satisfied with the work, some constraining factors were identified. A number of these constraints have also been identified in similar programs across the globe leading to reduced program impact. For the ongoing development and success of these programs, it is imperative to learn from the lessons of other program implementations and continue to evaluate new and ongoing programs and address any constraining factors that are identified. One significant area for improvement identified in this and other evaluations is to ensure that the work is supported by and integrated into local health services so that integrated care can be accessed by their disadvantaged populations.

## References

[1] Harris B, Fetherston H, Calder R. Australia’s Health Tracker by Socio-Economic Status (SES). Melbourne, Victoria: Australia Health Policy Collaboration, Victoria University 2017. [cited 2020 Oct 14]. Available from: https://www.vu.edu.au/sites/default/files/australia-health-tracker-by-ses-mitchell-institute.pdf.

[2] Kim K, Choi JS, Choi E, Nieman CL, Joo JH, Lin FR, Gitlin LN, Han HR. Effects of community-based health worker interventions to improve chronic disease management and care among vulnerable populations: A systematic review. American Journal of Public Health. 2016; 106(4): e3–e28. DOI: 10.2105/AJPH.2015.302987 PMC4785041 26890177

[3] Waisel DB. Vulnerable populations in healthcare. Current Opinion in Anesthesiology. 2013; 26(2): 186–92. DOI: 10.1097/ACO.0b013e32835e8c17 23385323

[4] Ali-Faisal SF, Colella TJ, Medina-Jaudes N, Benz Scott L. The effectiveness of patient navigation to improve healthcare utilization outcomes: A meta-analysis of randomized controlled trials. Patient Education & Counselling. 2017; 100(3): 436–48. DOI: 10.1016/j.pec.2016.10.014 27771161

[5] Mossabir R, Morris R, Kennedy A, Blickem C, Rogers A. A scoping review to understand the effectiveness of linking schemes from healthcare providers to community resources to improve the health and wellbeing of people with long-term conditions. Health & Social Care in the Community. 2015; 23(5): 467–84. DOI: 10.1111/hsc.12176 25494621

[6] Thomas L, Parker S, Song H, Gunatillaka N, Russell G, Harris M, Russell G, Haggerty J, Levesque J-F, Harris M, Dahrouge S, Lewis V, Scott C, Stocks N, on behalf of the Impact Team. Health service brokerage to improve primary care access for populations experiencing vulnerability or disadvantage: a systematic review and realist synthesis. BMC Health Services Research. 2019; 19(1): 269. DOI: 10.1186/s12913-019-4088-z 31035997 PMC6489346

[7] Javanparast S, Windle A, Freeman T, Baum F. Community health worker programs to improve healthcare access and equity: Are they only relevant to low- and middle-income countries? International Journal of Health Policy and Management. 2018; 7(10): 943–54. DOI: 10.15171/ijhpm.2018.53 30316247 PMC6186464

[8] Mistry SK, Harris E, Harris M. Community Health Workers as Healthcare Navigators in Primary Care Chronic Disease Management: a Systematic Review. J Gen Intern Med. 2021 (Epub ahead of print). DOI: 10.1007/s11606-021-06667-y PMC8390732 33674916

[9] Little TV, Wang ML, Castro EM, et al. Community Health Worker Interventions for Latinos With Type 2 Diabetes: a Systematic Review of Randomized Controlled Trials. Curr Diab Rep. 2014; 14: 558. DOI: 10.1007/s11892-014-0558-1 25374313 PMC6191032

[10] Zhou K, Fitzpatrick T, Walsh N, Kim JY, Chou R, Lackey M, Scott J, Lo YR, Tucker JD. Interventions to optimise the care continuum for chronic viral hepatitis: a systematic review and meta-analyses. Lancet Infectious Diseases. 2016; 16(12): 1409–22. DOI: 10.1016/S1473-3099(16)30208-0 27615026

[11] McBrien KA, Ivers N, Barnieh L, Bailey JJ, Lorenzetti DL, Nicholas D, et al. Patient navigators for people with chronic disease: A systematic review. PLoS ONE. 2018; 13(2): e0191980. DOI: 10.1371/journal.pone.0191980 29462179 PMC5819768

[12] Parekh TM, Copeland CR, Dransfield MT, Cherrington A. Application of the community health worker model in adult asthma and COPD in the U.S.: a systematic review. BMC Pulmonary Medicine. 2019; 19(1): 116. DOI: 10.1186/s12890-019-0878-7 31242944 PMC6593583

[13] Jack HE, Arabadjis SD, Sun L, Sullivan EE, Phillips RS. Impact of community health workers on use of healthcare services in the United States: A systematic review. Journal of General Internal Medicine. 2017; 32(3): 325–44. DOI: 10.1007/s11606-016-3922-9 27921257 PMC5331010

[14] Shommu NS, Ahmed S, Rumana N, Barron GRS, McBrien KA, Turin TC. What is the scope of improving immigrant and ethnic minority healthcare using community navigators: A systematic scoping review. International Journal for Equity in Health. 2016; 15(1): 6. DOI: 10.1186/s12939-016-0298-8 26768130 PMC4714538

[15] Valaitis RK, Carter N, Lam A, Nicholl J, Feather J, Cleghorn L. Implementation and maintenance of patient navigation programs linking primary care with community-based health and social services: a scoping literature review. BMC Health Services Research. 2017; 17(1): 1–14. DOI: 10.1186/s12913-017-2046-1 28166776 PMC5294695

[16] Woodall J, Trigwell J, Bunyan A-M, Raine G, Eaton V, Davis J, Hancock L, Cunningham M, Wilkinson S. Understanding the effectiveness and mechanisms of a social prescribing service: a mixed method analysis. BMC Health Services Research. 2018; 18(1): 604. DOI: 10.1186/s12913-018-3437-7 30081874 PMC6080378

[17] Cometto G, Ford N, Pfaffman-Zambruni J, Akl E, Lehmann U, McPake B, Ballard M, Kok, M, Najafizada M, Olaniran A, Ajuebor, O, Perry HB, Scott K, Albers B, Shlonsky A, Taylor D. Health policy and system support to optimise community health worker programmes: an abridged WHO guideline. The Lancet Global Health. 2018; 6(12): 1397–404. DOI: 10.1016/S2214-109X(18)30482-0 30430994

[18] Schmidt B, Campbell S, McDermott R. Community health workers as chronic care coordinators: evaluation of an Australian Indigenous primary health care program. Australian and New Zealand Journal of Public Health. 2016; 40(Suppl 1): S107–14. DOI: 10.1111/1753-6405.12480 26559016

[19] Lilley D, Standen C, Lloyd J. Healthy Waterloo: A study into the maintenance and improvement of health and wellbeing in Waterloo. (Final Draft). Sydney, Australia: Health Equity Research and Development Unit, UNSW; 2019.

[20] Sydney Local Health District. Year in review: 2018–19. Sydney: Sydney Local Health District; 2020. [cited 2020 Oct 16]. Available from: https://www.slhd.nsw.gov.au/pdfs/YIR_18-19.pdf.

[21] Counterpoint, RedWatch, Inner Sydney Voice, Sydney Local Health District. Report of the Waterloo Health Forum 2.0 Strategies for the improving the health and wellbeing of the residents of Waterloo now and in the future. Sydney, Australia; May 2018.

[22] Minkman M. Developing integrated care. Towards a development model for integrated care. International Journal of Integrated Care. 2012; 12(8). DOI: 10.5334/ijic.1060

[23] Braun V, Clarke V. Using thematic analysis in psychology. Qualitative Research in Psychology. 2006; 3(2): 77–101. DOI: 10.1191/1478088706qp063oa

[24] Corbin J, Strauss A. Basics of qualitative research (3rd ed.): Techniques and procedures for developing grounded theory. Thousand Oaks, CA: SAGE Publications; 2008. DOI: 10.4135/9781452230153

[25] Karmaz K. Constructing Grounded Theory. A practical guide through qualitative analysis. London: SAGE; 2006.

[26] QSR International Pty Ltd. (2018) NVivo (Version 12), https://www.qsrinternational.com/nvivo-qualitative-data-analysis-software/home.

[27] Wildman JM, Moffatt S, Steer M, Laing K, Penn L, O’Brien N. Service-users’ perspectives of link worker social prescribing: A qualitative follow-up study. BMC Public Health. 2019; 19(1): 98. DOI: 10.1186/s12889-018-6349-x 30670001 PMC6341763

[28] Sharma N, Harris E, Lloyd J, et al. Community health workers involvement in preventative care in primary healthcare: a systematic scoping review. BMJ Open. 2019; 9: e031666. DOI: 10.1136/bmjopen-2019-031666 PMC6937114 31852698

[29] Scott K, Beckham SW, Gross M, Pariyo G, Rao KD, Cometto G, Perry HB. What do we know about community-based health worker programs? A systematic review of existing reviews on community health workers. Human Resources Health. 2018; 16(1): 39. DOI: 10.1186/s12960-018-0304-x PMC6097220 30115074

[30] Pescheny JV, Pappas Y, Randhawa G. Facilitators and barriers of implementing and delivering social prescribing services: a systematic review. BMC Health Services Research. 2018; 18(1): 86. DOI: 10.1186/s12913-018-2893-4 29415720 PMC5803993

